# Short-latency afferent inhibition is a poor predictor of individual susceptibility to rTMS-induced plasticity in the motor cortex of young and older adults

**DOI:** 10.3389/fnagi.2014.00182

**Published:** 2014-08-07

**Authors:** Marielle Young-Bernier, Annick N. Tanguay, Patrick S. R. Davidson, François Tremblay

**Affiliations:** ^1^School of Psychology, University of OttawaOttawa, ON, Canada; ^2^Bruyère Research Institute, University of OttawaOttawa, ON, Canada; ^3^Canadian Partnership for Stroke Recovery, University of OttawaOttawa, ON, Canada; ^4^School of Rehabilitation Sciences, University of OttawaOttawa, ON, Canada

**Keywords:** aging, cholinergic function, theta burst, transcranial magnetic stimulation, short afferent inhibition

## Abstract

Cortical plasticity, including long-term potentiation (LTP)-like plasticity, can be assessed non-invasively with repetitive transcranial magnetic stimulation (rTMS) protocols. In this study, we examined age differences in responses to intermittent theta burst stimulation (iTBS) in a group of 20 young and 18 healthy older adults. Because the cholinergic system plays a role in the neural processes underlying learning and memory, including LTP, we also investigated whether short latency afferent inhibition (SAI), a neurophysiological marker of central cholinergic activity, would be associated with age-related differences in LTP-like plasticity induced by iTBS.

**Methods**: SAI was first assessed by examining the modulation of motor evoked potentials (MEPs) in response to median nerve conditioning 20 ms prior to TMS. Participants then underwent iTBS (3 pulses at 50 Hz every 200 ms for 2 s with 8 s between trains, repeated 20 times). MEP responses (120% resting motor threshold (RMT)) were assessed immediately after iTBS and 5, 10, and 20 min post-application.

**Results**: Responses to iTBS were quite variable in both age groups, with only approximately 60% of the participants (*n* = 13 young and 10 older adults) showing the expected facilitation of MEP responses. There were no significant age group differences in MEP facilitation following iTBS. Although older adults exhibited reduced SAI, individual variations were not associated with susceptibility to express LTP-like induced plasticity after iTBS.

**Conclusion**: Overall, these results are consistent with reports of high inter-individual variability in responses to iTBS. Although SAI was reduced in older adults, consistent with a deterioration of the cholinergic system with age, SAI levels were not associated with LTP-like plasticity as assessed with iTBS.

## Introduction

Non-invasive transcranial magnetic stimulation (TMS) can be used to explore the neurophysiological mechanisms underlying synaptic plasticity in the human motor cortex through various repetitive protocols (rTMS). One of these protocols, theta burst stimulation (TBS; Huang et al., 2005), has gained particular attention as it is relatively short in duration, uses a low intensity of stimulation, and can induce lasting changes in brain excitability that are very similar to those described in *in vitro* studies in terms of long-term potentiation (LTP) and long-term depression (LTD; Huang et al., 2007; Teo et al., 2007). When applied in an intermittent pattern, TBS (i.e., iTBS) generally leads to the facilitation of motor evoked potentials (MEPs) and induces LTP-like plasticity in the motor cortex.

Initial reports on iTBS revealed robust facilitation of brain excitability (Huang et al., 2005), but considerable inter-individual variability has more recently been described with up to 50% of participants not exhibiting the expected facilitation of MEP responses (e.g., Player et al., 2012; Hamada et al., 2013; Vallence et al., 2013; Hinder et al., 2014; López-Alonso et al., 2014). Factors such as genetics, voluntary motor activity, sex, and physical exercise all contribute to this variability (Ridding and Ziemann, 2010). Of importance to this study, aging has also been associated with a reduced modulation of brain excitability by TBS and other rTMS plasticity-inducing protocols, including paired associative stimulation (PAS; Müller-Dahlhaus et al., 2008; Tecchio et al., 2008; Fathi et al., 2010; Freitas et al., 2011). Only one study has examined age effects on iTBS responses in a small group of participants but, although a slight reduction in LTP-like plasticity with age was described, results were non-significant (Di Lazzaro et al., 2008b).

The deterioration of the cholinergic system in aging is thought to contribute to age-related changes in learning and memory due to the critical role of cholinergic innervations in modulating cortical plasticity and LTP-like processes (Rasmusson, 2000). Pharmacological studies have supported an effect of acetylcholine on responses to plasticity-inducing rTMS protocols. Indeed, cholinergic agonists, such as nicotine and the cholinesterase inhibitor rivastigmine, tend to increase and prolong facilitatory iTBS and PAS effects (Kuo et al., 2007; Swayne et al., 2009; Thirugnanasambandam et al., 2011; but see Korchounov and Ziemann, 2011). In contrast, the administration of a cholinergic antagonist to young adults reduces LTP-like plasticity following PAS (Korchounov and Ziemann, 2011). PAS-induced LTP-like plasticity is also reduced in Alzheimer’s disease (AD), which is often considered a model of chronic deficient central cholinergic activity (Battaglia et al., 2007). The effect of chronic age-related changes in cholinergic integrity on responses to iTBS, as opposed to the acute effects of cholinergic agonists and antagonists on acetylcholine’s levels in the brain, has not been examined in a healthy population.

Central cholinergic activity can be examined using TMS by applying a contralateral conditioning stimulation to the median nerve 18–20 ms prior to the TMS pulse. This pairing generally leads to the inhibition of MEPs and is called short-latency afferent inhibition (SAI; Di Lazzaro et al., 2000; Tokimura et al., 2000). SAI levels are significantly reduced by scopolamine, a muscarinic cholinergic antagonist, in young healthy adults (Di Lazzaro et al., 2000) and can be improved with acetylcholinesterase inhibitors in patients with AD (Di Lazzaro et al., 2002). Using a constant TMS test intensity protocol, we have previously shown that SAI is also reduced in normal aging (Young-Bernier et al., 2012; but see Oliviero et al., 2006; Degardin et al., 2011).

In this study, we investigated age-related differences in the modulation of cortical excitability following iTBS in young and healthy older adults. Given the cholinergic system’s role in the underlying processes supporting plasticity, we also examined whether SAI levels, as a neurophysiological marker of cholinergic activity, are associated with individual responses to iTBS and could explain part of the inter-individual variability in plasticity-inducing TMS protocols.

## Methods

### Participants

Young adults (*n* = 20; age range = 22.3 ± 3.2 years; 13 females) and healthy older adults (*n* = 18; age range = 70.1 ± 5.6 years; 9 females) were recruited from the local community (the participants included in this study are part of a larger cohort; their SAI levels and performance on measures of attention are described elsewhere). Participants were screened for psychiatric or neurological disorder and contraindications to TMS. Older adults also completed the Montreal Cognitive Assessment (MoCA; Nasreddine et al., 2005) to screen for possible mild cognitive impairment. Although some older adults scored below the recommended cutoff of 26 points (*n* = 4, MoCA scores of 23–25/30), they were deemed eligible for the study based on the interview and evidence that this cutoff may be too high (Rossetti et al., 2011). Participant’s medications were not altered for testing, with many older adults taking drugs related to vascular health (i.e., hypertension, statins cholesterol lowering drugs). None of the participants were taking neuroactive drugs such as neuroleptics, however one young adult and one older adult were taking antidepressants but their TMS data were within normal limits. Data from five additional young adults were not included in this report because they could not complete the TMS assessment for their resting threshold was particularly high making stimulation uncomfortable. The institutional Research Ethics Boards approved this study. Participants provided informed consent and received a minimal honorarium to defray expenses for their participation.

### MEP recordings: determination of the hotspot and resting motor threshold

MEPs were recorded using small auto-adhesive surface electrodes (Ag/AgCl, Kendall Medi-Trace™ 130) placed over the first dorsal interosseous (FDI) and abductor pollicis brevis (APB) muscles of the right hand in a belly-tendon montage. Electromyographic signals were amplified and filtered with a time constant of 0.03 s and a low-pass filter of 1 kHz (AB-621G Bioelectric amplifier, Nihon-Kohden Corp., CA 92610). Signals were digitized at a rate of 2 kHz (BNC-2090, National Instrument Corp. Austin, TX, USA) and relayed to a laboratory computer running custom software to control acquisition. TMS was administered with participants comfortably seated in a recording chair. Movements of the head were restrained with a U-shape neck cushion. Single pulse magnetic stimulation was delivered via a Magstim 200 stimulator (Magstim Co. Dyfed, UK) connected to a figure-of-eight coil (70-mm loop diameter). The coil was held approximately 45° in the mid-sagittal plane and the approximate location of the hand motor area on the left hemisphere was explored in approximately 1-cm steps until reliable MEPs could be evoked in the target muscle. This site was then marked with a circular sticker to ensure consistent coil positioning. The coil was held in place manually over the hotspot by the same experimenter (François Tremblay) for all participants. Following this procedure, the resting motor threshold (RMT) was determined for both the FDI and APB using the maximum likelihood strategy for estimating motor thresholds (Awiszus, 2003; TMS Motor Threshold Assessment Tool 2.0; Brain Stimulation Laboratory, Medical University of South Carolina, USA). This method has been shown to produce similar results to Mills and Nithi’s (1997) method, while requiring a smaller number of stimulations to determine the motor threshold (Mishory et al., 2004).

### Baseline measures of corticospinal excitability

Test TMS intensity was fixed at 120% RMT for both the FDI and APB muscles. Baseline MEP amplitude in the FDI was first determined for each participant by eliciting 15 MEPs at rest. The same procedure was followed for the APB muscle after completion of the SAI protocol. Trials for which unwanted muscle contractions were present were eliminated and repeated.

### Short Afferent Inhibition (SAI)

For SAI, we used a protocol similar to the one described by Tokimura et al. (2000) and Di Lazzaro et al. (2000). Conditioning afferent stimulation consisted in the application of 200 μs electrical pulses (S88 Stimulator, Grass Technologies, Astro-Med, Inc, West Warwick, RI 02893 U.S.A.) on the median nerve at an intensity just above the motor threshold to evoke a minimal visible twitch of the thenar muscles. SAI was induced by applying conditioning afferent stimulation 20 ms before the TMS pulse over the motor cortex (120% RMT_FDI_). This conditioning interval was shown to be optimal to evoke MEP inhibition in several studies (e.g., Tokimura et al., 2000; Fischer and Orth, 2011). Fifteen MEPs were elicited at the conditioned 20 ms interval and recorded from the FDI muscle.

### Intermittent Theta-Burst Stimulation (iTBS)

TBS was delivered via a Magstim Rapid^2^ stimulator (Magstim Co. Wales, UK) connected to a figure-of-eight coil (90-mm inside loop diameter). We followed the iTBS procedure described by Huang et al. (2005). The iTBS protocol consisted of three pulses delivered at a frequency of 50 Hz (i.e., 1 burst) and applied every 200 ms for 2 s (10 bursts). This was repeated every 10 s for a total duration of 190 s and 600 pulses. The effect of iTBS was assessed by monitoring changes in the APB because some participants had taken part in an earlier pilot study based on the PAS protocol in which the APB was the target muscle. Stimulation intensity was set at 80% APB active motor threshold (AMT). The latter was determined while participants exerted a light tonic contraction by pinching a soft exercise ball between their thumb and index finger (∼15–20% maximum contraction). Blocks of 15 MEPs at 120% RMT_APB_ were elicited immediately after and 5, 10, and 20 min post-iTBS and recorded from the APB muscle.

### Analysis of MEP data

Mean individual MEP responses for each condition (i.e., baselines, SAI, and post-iTBS intervals) were determined off-line by averaging the amplitude (peak-to-peak) and latency of each trial. Data for SAI and iTBS were expressed as percent of baseline MEP amplitude for the relevant target muscle (i.e., SAI = % MEP_Conditioned_ / MEP_Resting FDI_; iTBS response = % MEP_Interval_ _post-iTBS_ / MEP_Resting APB_). Based on previous studies (e.g., Hamada et al., 2013; Hinder et al., 2014; López-Alonso et al., 2014), responders to the iTBS protocol were defined as the individuals who exhibited the expected facilitation of normalized to baseline MEP responses (>100%) when responses across the four post-iTBS time intervals were averaged.

### Statistical methods

Independent *t*-tests, with adjusted *p*-values for multiple comparisons (i.e., *p* = 0.01) were used to examine differences in baseline measures of excitability between the two age groups. A repeated measure analysis of variance (ANOVA) was used to detect main effect and interactions with post-iTBS time intervals (0, 5, 10, and 20 min) as the within-subjects factor and age group (young vs. older adults) or response to iTBS (responders vs. non-responders) as the between-subjects factor. The Fisher’s Exact Test for a 2 × 2 contingency table was used to examine differences in the number of responders to the iTBS protocol (i.e., mean MEP amplitude post-iTBS >100%) in each group. We used *t-*tests to examine age group differences in SAI level. Pearson’s correlations were used to examine associations among SAI levels and responses to iTBS. Significance level was set at *p* = 0.05. Analyses were performed with SPSS version 21.0 (Chicago, IL, USA) and figures were prepared with GraphPad Prism version 5.00 (San Diego, CA, USA).

## Results

### Age differences in baseline measures of excitability

Baseline TMS measures of excitability in both age groups are reported in Table 1. Overall, older adults exhibited a trend towards decreased cortical excitability (e.g., elevated resting MT) but age-related differences were only significant in regard to an increased latency of MEP responses in seniors (*p* < 0.001).

**Table 1:** **Baseline measures of cortical excitability (mean ± SD) in the two age groups**.

	**Young adults (*n* = 20)**	**Older adults (*n* = 18)**
*Baseline measurements for iTBS (APB muscle)*		
Resting MT (% output)	43.0 ± 8.6	47.6 ± 10.8
MEP amplitude (μV)	560.9 ± 408.2	569.5 ± 484.6
MEP latency (ms)	21.3 ± 0.3	24.1 ± 2.8*
AMT (% output)	63.9 ± 11.1	64.1 ± 8.9
*Baseline measurements for SAI (FDI muscle)*		
Resting MT (% output)	42.6 ± 9.3	46.2 ± 11.2
MEP amplitude (µV)	898.2 ± 826.5	507.6 ± 484.9
MEP latency (ms)	21.0 ± 1.6	24.4 ± 3.0*
Intensity MNS (V)	6.3 ± 2.1	7.3 ± 1.8

*Abbreviations: AMT: active motor threshold; APB: abductor pollicis brevis; FDI: first dorsal interosseous; iTBS: intermittent theta burst stimulation; MEP: motor evoked potential; MNS: median nerve stimulation (intensity of); MT: motor threshold; SAI: short latency afferent inhibition*.

* Significant difference at adjusted *p*-value (*p* = 0.01) for multiple comparisons.

### Responses to iTBS and age differences

**Figure 1:**
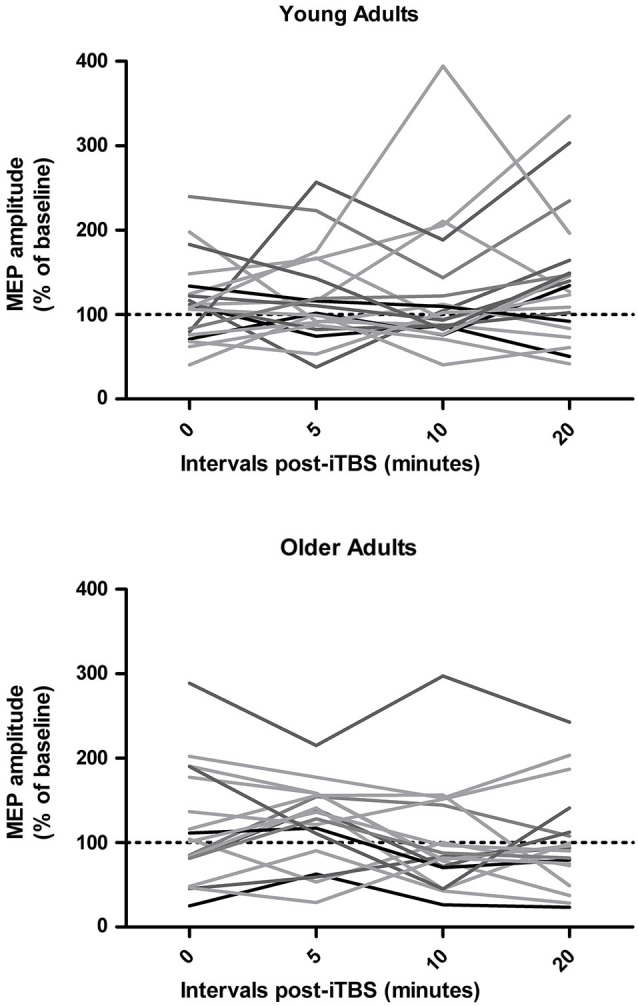
**Individual responses to intermittent theta burst stimulation (iTBS) in young and older adults at 0, 5, 10, and 20 min post-iTBS**.

As shown in Figure 1, inter-individual responses to iTBS were quite variable. Mixed ANOVAs revealed no main effect of time interval on iTBS responses in the young (*F*_3,57_ = 0.97, *p* = 0.41) and older adults (*F*_3,51_ = 1.78, *p* = 0.16). Overall changes in MEP amplitude ranged from −40% to 218% in young adults and from −65% to 261% in older adults. Only 13/20 young adults (65%) and 10/18 older adults (56%) exhibited the expected increase in MEP amplitudes following iTBS when their responses across the four time intervals were averaged (i.e., %MEP_post-iTBS_ > 100%). A Fisher’s exact test analysis did not reveal any difference in the ratio of responders in young and older adults (*p* = 0.74). Baseline RMT and AMT were negatively associated with overall iTBS responses in older adults (*r* = −0.55 and −0.74, respectively; *p* < 0.02) but not in young adults (*r* < −0.41), while there were no significant correlations with baseline MEP latency.

**Figure 2:**
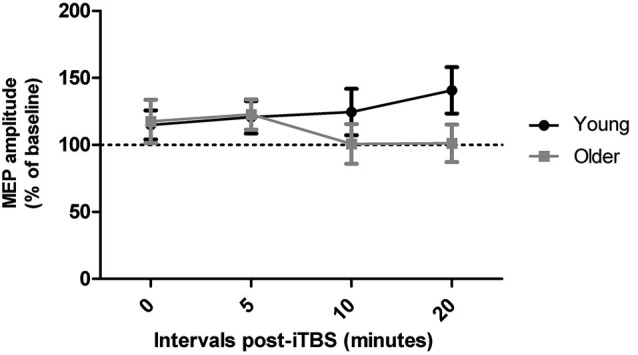
**Effect of iTBS at times 0, 5, 10, and 20 min following the procedure in young and older adults**.

Qualitatively, both age groups exhibited MEP facilitation after iTBS and there was a trend for MEP amplitudes to return to baseline levels more quickly in older adults (after approximately 10 min) than in young adults, who were still showing facilitation 20 min post-iTBS (Figure 2). However, these group differences did not reach significance as a repeated measures ANOVA did not reveal a main effect of time (*F*_3,108_ = 0.36, *p* = 0.79) or age group (*F*_1,36_ = 0.80, *p* = 0.38) on iTBS responses and time interval by group interaction (*F*_3,108_ = 2.08, *p* = 0.11). An additional repeated measures ANOVA did not yield any main effect or interaction between time and age group when only the responders to iTBS were included in the analysis (*n* = 23, *p* > 0.14).

### Variations in SAI in relation to responses to iTBS

**Figure 3:**
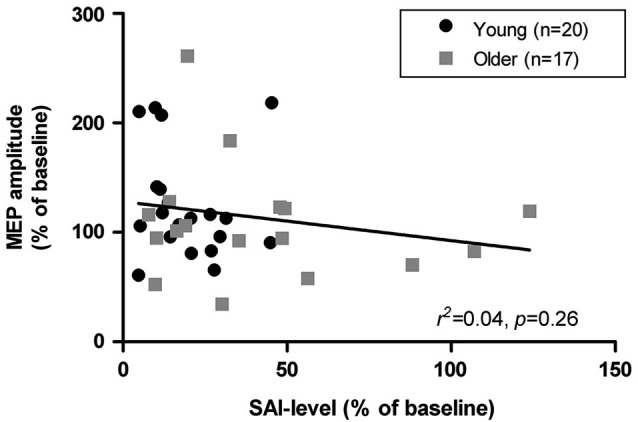
**Association between short latency afferent inhibition (SAI) levels and mean modulation of MEP amplitudes (time intervals 0, 5, 10, and 20 min) following iTBS in young and older adults**.

Data from one older adult was excluded from all analysis involving SAI due to an abnormally high MEP facilitation in response to afferent conditioning (Grubb’s test, *p* < 0.01, *z* = 3.32). Young adults exhibited significantly deeper levels of SAI than older adults (19.43 ± 12.13% vs. 42.07 ± 34.79%, respectively; *p* = 0.01). SAI measures in the older group were also characterized by higher variability with four seniors showing either low or absent inhibition (i.e., SAI ≥ 50%) and two even showing facilitation in response to afferent conditioning. As illustrated in Figure 3, age-related variations in SAI levels were only poor predictors of corresponding variations in mean responses to iTBS. Similar non-significant associations were found when both groups were examined separately (young: *r*^2^ = 0.02, *p* = 0.61; older adults: *r*^2^ = 0.03, *p* = 0.54). SAI levels were also weakly associated with iTBS responses at the various time-intervals examined within the young and older adults (*r* < −0.37, *p* > 0.05). There were no significant differences in SAI levels between responders and non-responders to iTBS in both the young (*t*_18_ = 1.30, *p* = 0.21) and older groups (*t*_15_ = 0.67, *p* = 0.52). Furthermore, mean MEP modulation after iTBS in the four older adults with low SAI levels was similar to the other seniors (SAI < 50%). However, this subgroup of older adults exhibited less facilitation 5-min post-iTBS, but this effect was not present at the other test intervals.

## Discussion

In this study, we examined age-related differences in LTP-like plasticity induced by iTBS and their relationship with SAI, a marker of cholinergic activity in the motor cortex. Three main observations emerged from our results. First, individual responses to iTBS were quite variable as only 60% of participants exhibited the expected pattern of MEP facilitation. Second, there were no significant age group differences in iTBS-induced facilitatory effects. Finally, while older adults exhibited lower levels of SAI than younger adults, age-related variations in SAI were not associated with individual susceptibility to iTBS-induced plasticity. The significance of our results and possible confounding factors are discussed below.

### iTBS: variability and aging

Consistent with recent reports of large inter-individual variability in iTBS responses (e.g., Player et al., 2012; Hamada et al., 2013; Vallence et al., 2013; Hinder et al., 2014; López-Alonso et al., 2014), we found that susceptibility to express LTP-like plasticity following iTBS was variable in both young and older adults. Only about 60% of participants displayed the expected overall facilitation of MEP amplitudes and the number of responders to iTBS was similar in both age groups (i.e., 65% of young adults and 58% older adults). Accordingly, we did not find a significant difference between young and older adults in terms of MEP facilitation following iTBS, even after sorting participants to retain only the “responders” (mean post-iTBS modulation >100%). In this respect, our results are in line with those of Di Lazzaro et al. (2008b) who also examined age effects on responses to iTBS in a small sample of participants but did not find LTP-like plasticity to be significantly reduced in older adults. In contrast, cTBS has been shown to lead to smaller and shorter lasting inhibitory effects in aging (Freitas et al., 2011) and may thus be more reliably affected by age. We did not find significant age differences in the duration of iTBS effects in this study, but given that the young adults were still displaying MEP facilitation at 20 min, it is possible that an effect of age would have been found if we had examined later time intervals. However, this seems unlikely given that peak MEP modulation seems to occur between 5 and 20 min post-iTBS in healthy young adults (e.g., Swayne et al., 2009; Li Voti et al., 2011; Player et al., 2012; Cárdenas-Morales et al., 2014; López-Alonso et al., 2014; but see Hinder et al., 2014).

The other TMS studies that have investigated age effects on LTP/LTD-like plasticity have relied on the PAS protocol (Müller-Dahlhaus et al., 2008; Tecchio et al., 2008; Fathi et al., 2010). In general, these studies have reported significant age-related reductions in LTP-like plasticity after PAS, but differences between stimulation protocols may in part explain why we did not find similar age differences with iTBS. Indeed, both protocols do not attempt to produce plasticity in the same way: iTBS relies on low intensity bursts of stimulation and simulates natural cortical theta and gamma rhythms (Cárdenas-Morales et al., 2010) while PAS is dependent upon Hebbian synaptic strengthening following the synchronous activation of neurons by nerve and TMS stimulations (Stefan et al., 2000). Recent studies have also reported small correlations between MEP modulation by iTBS and facilitatory PAS, suggesting that the mechanisms on which they rely to induce LTP-like plasticity, while both being dependent on NMDA receptors, may only partially overlap (Player et al., 2012; Vallence et al., 2013; López-Alonso et al., 2014).

Additionally, in these studies, the test and stimulation intensity was based on MEP size, a procedure that can lead to overstimulation (Garry and Thomson, 2009), especially in seniors. In the present study, the stimulation intensity was based on individual motor thresholds, which could have contributed to mitigate age differences in responses to iTBS. In agreement with this, we found negative correlations between overall iTBS responses and RMT and AMT in seniors, suggesting that stimulation intensity can influence the magnitude of MEP modulation by rTMS. However, as others, we did not find significant age differences in these measures, suggesting that cortical atrophy (and therefore increased distance between the coil and stimulation site) did not significantly contribute to reduced cortical plasticity in aging (Fathi et al., 2010; Freitas et al., 2011; but see Müller-Dahlhaus et al., 2008). Although we found age differences in the latency of baseline MEPs, these were not associated with iTBS responses. Still, it remains possible that the stimulation intensity used for our iTBS protocol was not optimal and this might explain why the overall effect of iTBS on motor cortical excitability was not significant in our study, which is in contrast to previous reports (e.g., Huang et al., 2005; Iezzi et al., 2008). On the other hand, the intensity of stimulation might not be the sole explanatory factor as other recent reports addressing the issue of inter-individual variability in responses to rTMS protocols have used the constant MEP size approach to monitor changes in excitability, and yet, they also failed to report a significant iTBS effect in young adults (Player et al., 2012; Hamada et al., 2013; Vallence et al., 2013; López-Alonso et al., 2014). Clearly, there is a need to better delineate the influence of TMS parameters on the variability in responses to plasticity-inducing protocols.

Many other factors have also been shown to influence responses to TBS. Of particular interest, voluntary muscle activity before or during TBS (Huang et al., 2008) can reverse and abolish plasticity effects, while contractions immediately after iTBS leads to greater MEP modulation (Huang et al., 2008; Iezzi et al., 2008). These studies suggest that baseline cortical excitability might greatly influence TBS and should thus be monitored more carefully in future studies. Controlling for other factors such as genetics (Cheeran et al., 2008; but see Li Voti et al., 2011; Mastroeni et al., 2013) and diurnal variations related to the time of day the testing took place (Sale et al., 2007; but see López-Alonso et al., 2014) may also prove useful in maximizing the characterization of cortical plasticity as induced by TBS in aging. Another interesting line of questioning was raised by a recent study, which suggests that high inter-individual variability in TBS responses might be more closely related to differences in the population of interneurons recruited by rTMS than to intrinsic neuronal capacity for plasticity (Hamada et al., 2013). Indeed, when late I-waves were easily recruited by an anterior-posterior directed TMS current, participants were more susceptible to exhibit long-lasting TBS responses. The differential recruitment of interneurons as described by Hamada et al. (2013) accounted for approximately 50% of the variability in TBS responses. Whether similar effects may also be found in aging should be investigated.

### Relationship between iTBS and SAI

The main contribution of this study relates to our observation of a null correlation between individual responses to iTBS and SAI levels, a marker of central cholinergic activity in the motor cortex. This lack of relationship was present despite clear reductions in SAI with age, which is consistent with our previous results in an independent sample (Young-Bernier et al., 2012; but see Oliviero et al., 2006; Degardin et al., 2011) and other evidence of a deterioration of the cholinergic system in normal aging (e.g., Mesulam et al., 2004; Duzel et al., 2010; Dumas and Newhouse, 2011; Grothe et al., 2012).

Both iTBS and SAI have been shown to be preferentially dependent upon the recruitment of late I-waves, suggesting that they both involve similar cortical circuits (Di Lazzaro et al., 2004, 2008b). Accordingly, SAI levels can be increased and partially restored by cTBS in patients with AD, but they are not modulated by iTBS or PAS in young and healthy older adults (Di Lazzaro et al., 2011; Di Lorenzo et al., 2013). A significant decline in cholinergic activity may thus need to be present for strong associations between cortical plasticity and SAI levels to be present. Such potential effects could have been masked by the fact that only four of the older adults included in this study exhibited an absence or reduction of cortical inhibition (SAI ≥ 50%). Although this subgroup did not differ from the other older adults in regards to mean levels of MEP modulation following iTBS, they tended to exhibit less facilitation at 5 min post-iTBS. This could suggest that cholinergic activity contributes to the early build-up of cortical plasticity. We might be able to shed more light on this issue and find the expected relationship between SAI and LTP-like plasticity in a larger group of older adults with reduced SAI levels or if we investigated TBS effects in patients with AD, vascular damage or other diseases in which the deterioration of cholinergic transmission is a major pathologic component.

Our results suggest that age-related declines in cholinergic activity (as indexed by SAI) do not significantly contribute to the modulation of MEP amplitudes by iTBS or to the duration of these effects. These results are in contrast with pharmacological studies that have demonstrated increased and prolonged iTBS and PAS induced LTP-like plasticity following the administration of cholinergic agonists but reduced effects by antagonists (Kuo et al., 2007; Swayne et al., 2009; Korchounov and Ziemann, 2011; Thirugnanasambandam et al., 2011). Discrepancies between the results of these studies and ours might be related to our use of SAI levels to examine chronic age-related changes in cholinergic activity as opposed to the effects of phasic alterations in cortical cholinergic availability by drugs. As discussed above, these differences could also be related to the fact that most of these pharmacological studies were performed with PAS as opposed to iTBS (i.e., Kuo et al., 2007; Korchounov and Ziemann, 2011; Thirugnanasambandam et al., 2011). In this regard, Kuo et al. (2007) argued that rivastigmine leads to more LTP-like plasticity following PAS than transcranial direct current stimulation (tDCS) due to acetylcholine’s role in increasing the signal-to-noise ratio and enhancing the detection of afferent sensory inputs. The influence of iTBS appears to be more diffuse than the synapse-specific PAS effects and different influences of the cholinergic system on LTP-like responses could thus be expected following each of these protocols.

Additionally, declines in cholinergic activity are only one of many changes taking place in central neurotransmission during normal aging (e.g., Yankner et al., 2008) and may thus not be sufficient to predict age-related differences in responses to plasticity-inducing rTMS protocols. For example, age-related changes in dopamine (Bäckman et al., 2006) may also contribute to the modulation of cortical plasticity (Kuo et al., 2008; Korchounov and Ziemann, 2011).

Finally, although there is ample reason to think of SAI as a good marker of cholinergic activity (SAI is reduced by scopolamine in healthy adults (Di Lazzaro et al., 2000), it is decreased in patient populations with deficient cholinergic activity (e.g., AD, Lewy Body disease, vascular dementia; Di Lazzaro et al., 2007b, 2008a), and it can be rescued by acetylcholinesterase inhibitors in AD patients (Di Lazzaro et al., 2002), SAI may also be modulated at the cortical level in part by GABA_A_ receptors. The efficiency of this motor cortical inhibition circuit is influenced by benzodiazepines (Di Lazzaro et al., 2007a), consistent with a role of the GABAergic system in controlling acetylcholine release in the cortex (Giorgetti et al., 2000). Also, SAI is decreased in patients with obsessive-compulsive disorder (Russo et al., 2014) and posttraumatic stress disorder (Rossi et al., 2009), which are both psychiatric conditions involving a GABAergic imbalance but with limited cholinergic involvement. The age-related reductions in SAI described in this study may thus reflect not only declines in cholinergic activity but also alterations in GABAergic transmission, though this system appears to be relatively spared in aging (Rissman et al., 2007).

In summary, the present study provides further evidence of high inter-individual variability in responses to iTBS in both young and healthy older adults. We also found evidence of declines in SAI levels with age, but these were not associated with iTBS responses. Together, these results suggest that chronic changes in cholinergic neuromodulation as they occur in normal aging do not significantly contribute to the inter-individual variability in LTP-like plasticity induced by iTBS.

## Authors’ contributions

Marielle Young-Bernier participated in the design of the study, carried out the data collection, analyzed the data, and drafted the manuscript. Annick N. Tanguay participated in the collection and analysis of data. Patrick S. R. Davidson participated in the design of the study and in editing the manuscript. François Tremblay conceived the study, aided with data collection and in drafting and editing the manuscript. All authors read and approved the final manuscript.

## Conflict of interest statement

The authors declare that the research was conducted in the absence of any commercial or financial relationships that could be construed as a potential conflict of interest.
